# Supplementary figures and images for: Structural insight into the stabilization of microtubules by taxanes
Source: eLife. 2023 Mar 6;12:e84791. doi: 10.7554/eLife.84791 (PMC10049219; doi:10.7554/eLife.84791)

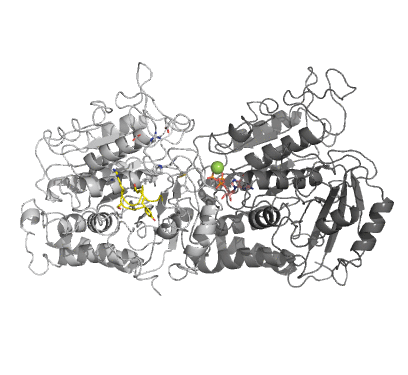

Supplement: Supplementary file 5 [file elife-84791-animation1.gif]

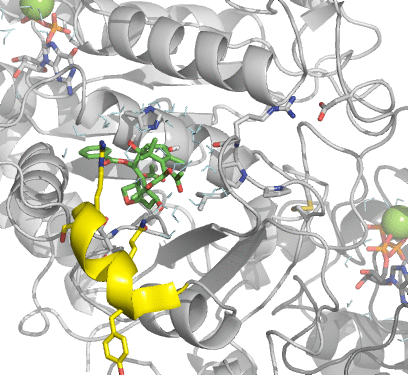

Supplement: Supplementary file 6 [file elife-84791-animation2.gif]

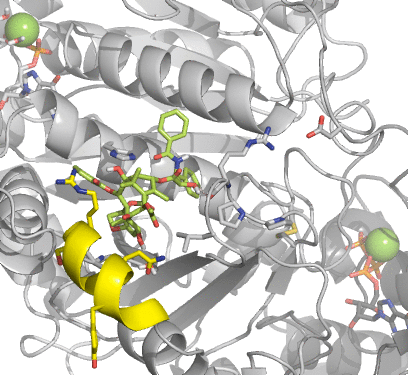

Supplement: Supplementary file 7 [file elife-84791-animation3.gif]
